# Examining clinical relevance of interpretation inflexibility in social contexts

**DOI:** 10.1186/s12991-026-00683-0

**Published:** 2026-06-20

**Authors:** Wisteria Deng, Reut Zabag, Saumya Narain, Tyrone D. Cannon, Jutta Joormann

**Affiliations:** 1 Department of Psychology, Yale University, 100 College Street, New Haven, CT 06520 USA; 2 Department of Psychiatry, Yale University, New Haven, USA

**Keywords:** Interpretation Inflexibility, Bias, Social Processing, Psychosis, Depression, Belief Revision

## Abstract

Growing research highlights interpretation inflexibility as a key transdiagnostic mechanism across psychopathologies. Yet, few studies have examined its role in everyday socio-emotional processing. This review bridges that gap by exploring how interpretation inflexibility contributes to psychopathology—particularly depression and psychosis—in social contexts. Evidence suggests disrupted interpersonal processes, including rigid interpretations of social scenarios and impressions of others, are central to understanding depressive and psychotic symptoms. Depression is marked by valence-specific, context-dependent inflexibility, with difficulty disengaging from negative biases. In contrast, psychosis shows a broader inflexibility across emotional valences. The review also outlines evolving measures of interpretation inflexibility, emphasizing the need to account for social context in interpretation revision. Current findings highlight the value of examining inflexibility within social frameworks to better understand its role in adaptive functioning and psychopathology. Future research on interpretation inflexibility in social contexts could inform novel interventions and improve clinical outcomes.

## Introduction

Exposure to ambiguous information is part of everyday life. The process of disambiguating this information is referred to as interpretation. Interpretation is prone to bias, which is the tendency to perceive ambiguous information as either positive or negative [1]. Bias at the interpretation stage may affect one’s ability to adapt to changing circumstances [2] and is associated with a multitude of psychological disorders including depression [3], anxiety [4], and psychosis [5]. Interpretation biases in social situations and when processing social cues may be especially problematic as social situations and cues are often highly ambiguous. Interpretations play a key role in making sense of social situations – perceiving them as threatening, neutral, or positive. Not surprisingly, interpretation biases have been associated with social functioning impairments [6, 7].

Most studies on interpretation biases have focused on identifying the presence of these biases [8]. Recent work, however, has shown that in addition to biases, the flexibility with which these biases are held and revised when disconfirming evidence is encountered, is a critical process for our understanding of psychopathology ([9, 10]. Bias in interpretation and belief inflexibility – defined as an inability to disengage from existing interpretations and form new beliefs when new information becomes available – has emerged as another mechanism implicated in the maintenance of depression, anxiety and psychotic disorders (e.g., delusional beliefs and conviction [11]. While numerous reviews have examined interpretation biases and their clinical implications [8], the role of inflexibility has yet to be examined in-depth, especially in relation to the processing of social situations. This review aims to fill in this gap by examining research conducted in both clinical and social psychological study contexts to better understand how interpretation inflexibility play a role in psychological disorders.

Research in social psychology has found that the ability to flexibly re-interpret social information is highly important for social functioning [12]. Flexibly updating social interpretations allows individuals to revise impressions of others’ characters, promote interpersonal relationship cohesion and aid interpersonal decision-making (e.g., whether to seek friendship or distance oneself) [13, 14]. Inflexible negative interpretations of other individuals’ action may discourage one’s future social engagement with those others, resulting in isolation, withdrawal, and increased negative affect [15, 16]. At the same time, negative affect and decreased interest in interpersonal relationships, often referred to as social anhedonia [17], decrease one’s motivation to flexibly revise interpretations of social situations [18]. On a societal level, flexibly revising interpretations of social stimuli plays an integral role in facilitating social cohesion and reducing stereotypes (i.e., fixed beliefs about certain social groups). Overall, given that social stimuli are often highly ambiguous, the interpretations of these stimuli may vary drastically and can have direct downstream consequences related to social functioning [6]. It is thus important to explore the ability to revise these stimuli in relation to functional and clinical outcomes.

To elucidate the role of interpretation inflexibility, the review will (1) consider the importance of interpretation bias and inflexibility in understanding psychopathology, (2) introduce tasks that assess interpretation inflexibility in social contexts, and (3) review clinical implications of inflexibility in social contexts. Four electronic databases (Web of Science, PsycINFO, MEDLINE, and PubMed) were searched on 1st December 2022 with no date restrictions. The search was rerun on 15th January 2025 to identify new publications since the first search.

## Bias and inflexibility in psychopathology

Interpretation bias has garnered significant attention as a transdiagnostic mechanism, cutting across a range of psychiatric disorders including social anxiety [66], eating disorders [19], depression [20], and psychosis [21].

Psychosis research has long provided a primary framework for examining interpretation inflexibility and bias, with consistent evidence linking these processes to multiple dimensions of delusion severity [20, 22]. Meta-analytic findings indicate that interpretation inflexibility is most strongly associated with delusional conviction, with smaller but reliable effects on preoccupation and distress [11]. Individuals with active delusions often not only discount the possibility of being mistaken, but also fail to generate or consider alternative explanations for their experiences [23]. Importantly, this tendency appears to extend beyond delusional content: patients exhibit similar resistance to revising neutral or unrelated beliefs, suggesting a generalized rigidity in reasoning style rather than a belief-specific deficit [24]. However, this inflexibility is not uniform. Experimental work shows that individuals with schizophrenia update weakly held beliefs comparably to controls, but demonstrate pronounced resistance when beliefs are strongly endorsed, indicating that belief strength moderates flexibility [25]. Longitudinal evidence further suggests that these reasoning biases remain stable over time, even as symptom severity fluctuates, pointing to their role as enduring cognitive features rather than transient clinical states [26].

Within this broader context, paranoia represents a particularly well-characterized and methodologically tractable manifestation of interpretation inflexibility. A comprehensive systematic review found that both clinical and subclinical paranoia are associated with elevated interpretation biases relative to controls [21]. These biases rarely operate in isolation; rather, they co-occur with other maladaptive reasoning tendencies, including jumping-to-conclusions (JTC), externalizing attributional styles, and belief inflexibility [22, 26]. Together, these processes appear to form a self-reinforcing cycle: threat-biased interpretations of ambiguity heighten perceived danger, strengthen paranoid beliefs, and further entrench biased information processing [21]. Notably, the presence of these biases prior to illness onset suggests that they may function as vulnerability markers, rather than simply emerging as consequences of psychosis [27].

Among these research efforts, there has also been an especially abundant amount of literature examining interpretation bias in depression, drawing a long-established connection between bias and depressive symptoms [1, 7, 28]. In particular, the tendency to make negative interpretations is attributed to mood-congruent cognitive bias [29], which suggests that individuals with depressive symptoms are more likely to resolve ambiguity in negative ways [28], congruent with their negative affective states. Most studies that utilized self-report measures have found that participants diagnosed with depression interpret ambiguous information in negative ways. For example, the Ambiguous Scenario Test – Depression (AST-D) prompts participants to form interpretations about open-ended scenarios, such as “It is New Year’s Eve. You think about the year ahead of you”. People with depressive symptoms tend to report less pleasantness associated with this kind of mental imagery, underlining the negative biases in interpreting ambiguous situations. Another widely used measurement for interpretation biases involves similar open-ended scenarios. Participants are asked to fill in words that complete the scenarios in either a negative or neutral way. For example, “The Doctor examined little Emily’s growth” is paired with a negative interpretation (target word: tumor) and a neutral interpretation (target word: growth). Interpretation biases are then captured by measuring response latency – participants exhibit a negative interpretation bias when they are faster at endorsing negative target words as compared to neutral ones. With these tasks, researchers have established a strong association between interpretation biases and depression at varying levels of severity.

Interpretation biases play an important role in both the emergence and maintenance of depression. The cognitive theory of depression postulates that biased interpretation of emotional information is a risk factor of depression. Cognitive vulnerability theories of depression argue that those most at risk can be characterized by how they explain the cause and consequence of negative events, which reflect their vulnerability for depression. In particular, a negative cognitive style may involve a pattern of making attributions that are internal, stable, and global (as opposed to external, temporary, or specific), assuming negative events will have further negative consequences, and making negative inferences about oneself, such as assuming the events that have occurred are a reflection of the self. Distorted cognitive processes like these have been found not only in individuals with active depression, but also in individuals at-risk for the disorder and individuals in remission. There is also evidence of negative interpretation biases specifically appearing in never-depressed daughters of mothers with depression, which provides further evidence that these biases may be a risk factor for the onset of this disorder. Interpretation biases have also been associated with higher levels of rumination, worry, and negative intrusive thoughts. Rumination and worry are forms of repetitive negative thinking common to depression, and interpreting ambiguous information in a negative manner may maintain these repetitive negative thinking patterns. Taken together, interpretation biases play a role in the vulnerability to, severity and maintenance of depression.

The work just reviewed has provided substantial theoretical background for designing interventions focused on bias modification. Multiple such bias modification interventions have targeted negative interpretations of ambiguous stimuli by giving positive feedback when individuals select a positive interpretation of a picture or a sentence, and giving negative feedback (e.g., “wrong answer”) when individuals make negative interpretations [7, 30, 31]. These bias modification interventions are widely discussed as reducing symptoms of social anxiety and depression [7, 8]. Numerous meta-analyses and treatment studies [31] have underlined the effectiveness of treatment modifications in boosting positive affect, encouraging adaptive emotion regulation and reducing depressive symptoms [32].

Overall, abundant literature has linked interpretation bias with the maintenance and treatment of clinical disorders [31]. However, these studies have mainly focused on the existence of such interpretation biases. Interpretation tendencies that are persistent and applied cross-situationally may arise and be maintained at least in part because of inflexibility in revising interpretations when encountering evidence contra-indicating these interpretations. Research has yet to adequately explore processes associated with inflexibility in revising such biases, which could represent an important transdiagnostic factor underlying symptoms above and beyond the effects of interpretation bias.

## Research on interpretation inflexibility as a transdiagnostic mechanism

At its very core, interpretation inflexibility impedes an individual’s ability to adaptively integrate novel and disconfirming information in response to evolving social situations. Such difficulties integrating new evidence and adapting to situational demands have been linked with depression through a few mechanisms. First, the rigidity in switching from emotional to non-emotional facial interpretations relates to depressive symptoms. used an affective shifting task requiring participants to sort facial expressions based on emotional and non-emotional content. Researchers found that participants with higher depressive symptoms exhibited greater difficulty switching from emotional to non-emotional tasks. This finding suggests that individuals with more severe depressive symptoms demonstrate inflexibility in disengaging from emotional stimuli. Second, difficulties regulating emotions, particularly shifting from negative to positive emotions, have been associated with depression. The use of less adaptive emotion regulation strategies, such as dampening, is linked with rigid and persistent negative interpretations of daily experiences. In particular, individuals with depressive symptoms tend to focus on interpreting the negative aspects of a situation and downplay the positive aspects. The same rigidity also relates to difficulties implementing adaptive emotion regulation strategies such as reappraisal and to the tendency to engage in repetitive negative thinking (e.g., rumination) [10]. Through these emotion regulation strategy selections, individuals experience a persistence in negative affect and feelings of hopelessness, further maintaining their depressive symptoms [33]. Third, the construct of ‘cognitive immunization’ may further explain the link between interpretation inflexibility and depressive symptoms. Cognitive immunization is defined as the reappraisal of expectation-disconfirming information in a way that maintains the existing expectations. In the context of depression, as individuals come across positive evidence contradicting their existing negative beliefs of the world, they may cognitively devalue such positive information - further perpetuating the existing negative biases and expectations. The same negative mood congruency that underlies negative bias in depression may thus play a crucial role in inflexibility, by dismissing or devaluing the disconfirmatory positive information. Overall, recent work has increasingly associated interpretation inflexibility with depressive symptoms.

In psychosis research, bias and inflexibility have been extensively studied through specific reasoning mechanisms such as JTC and impaired evidence integration, both of which have been implicated in delusion formation and persistence. found in their meta-analysis that JTC, alongside the bias against disconfirmatory evidence (BADE), bias against confirmatory evidence (BACE), and liberal acceptance are not uniform characteristics of schizophrenia, rather they are specifically tied to active delusional states. Patients with active delusions showed greater bias on all four measures than those in remission, while patients with schizophrenia with no current delusions showed no more JTC than healthy controls, consistent with the view that these biases are more closely linked to delusions than to the diagnosis itself. While JTC drives delusion formation by encouraging premature conclusions on insufficient evidence, BADE perpetuates them by diminishing sensitivity to disconfirming information. Extending on this, elucidated the two distinct components of BADE- integration of evidence and conservatism, out of which only evidence integration differed between severely delusional patients and all other groups. This sheds light on a specific deficit in incorporating new, disambiguating information, with the authors suggesting that individuals experiencing severe delusions tend to give excessive weight to evidence that confirms an existing belief, making it harder to revise that belief when contradictory information emerges. Another study supported this claim, finding moderate evidence linking belief inflexibility to delusional conviction, and for JTC when assessed with data-gathering tasks [22].

Recent work has begun to explore why these biases arise, with dual-process frameworks potentially offering some answers [15]. found that lower engagement in analytic reasoning was associated with greater BADE, partially explaining the link between reasoning deficits and persecutory ideation. Interestingly, the belief revision deficit was not limited to emotionally charged material but extended across neutral scenarios, pointing to a general impairment in updating beliefs in response to new evidence [15]. This serves as a meaningful distinction as we compare it with the pattern seen in depression, where inflexibility tends to be valence specific, tied to negatively valanced, self-referential material rather than reflecting a generalized difficulty with belief revision. A study directly comparing schizophrenia-spectrum and MDD outpatients found that reduced belief flexibility was specific to patients with active delusions, while explanatory flexibility did not differ between the two clinical groups [20]. Together, these findings suggest that while inflexible thinking is a shared feature across conditions, the locus differs: in psychosis, it cuts across emotional valence and disrupts the process of evidence integration itself; in depression, the content of rigid, fixed beliefs is the more defining characteristic.

While research linking interpretation inflexibility with depressive and psychotic symptoms is especially extensive, interpretation inflexibility has also been linked with other clinical diagnoses, such as eating disorders and social anxiety. Inflexible negative interpretation bias has been associated with significant emotion regulation difficulties and enhanced sensitivity to rejection – both mechanisms that perpetuate eating disorders, particularly anorexia nervosa [19]. Similarly, recent studies indicate that people with social anxiety consistently exhibit inflexible negative interpretations of environmental cues [15]. Engaging in such persistent negative interpretations engenders a sense of anticipated social failure and perceived social inadequacy, further exacerbating social anxiety.

## Measuring interpretation inflexibility

Taken together, interpretation inflexibility has been highlighted as a transdiagnostic mechanism, with abundant research expanding upon the early literature on interpretation bias and depression and psychosis. While there is a wealth of measures assessing interpretation bias [34], assessment of interpretation inflexibility as a dynamic process (of revisioning) is limited. Given the clinical relevance of interpretation inflexibility, it is crucial to examine tasks that assess inflexibility in empirical research.

Early measures of interpretation inflexibility were developed by psychosis researchers to evaluate cognitive rigidity [35], a factor underlying several psychotic symptoms such as persecutory beliefs and paranoia [36]. Examining these existing measures of inflexibility and their utility in psychosis research serves as a starting point to applying the same tasks across a broader diagnostic spectrum. For example, the Maudsley Assessment of Delusions Scale (MADS) evaluates eight key dimensions of delusional experiences. The belief maintenance section, particularly the Possibility of being Mistaken (PM) and the Reaction to Hypothetical Contradiction (RTHC) items were used to probe the participants about adjusting their delusional beliefs and viewing them as inaccurate [26]. Following this, the interviewer presented a plausible yet contradictory piece of evidence. As a proxy for interpretation flexibility, participants were measured on their ability to adjust their beliefs in response to the presented counterevidence. This popular clinical interview protocol was used to assess interpretation inflexibility in prominent large-scale studies, yielding findings that 24–57% of individuals with non-affective psychosis demonstrate a significant degree of inflexibility, as evidenced by PM and RTHC scores [23]. Another tool used to assess interpretation flexibility in psychosis was the Explanation of Experiences assessment (EoE), which measures the participants’ ability to generate alternative explanations for the evidence supporting their delusions [23]. Under a dichotomous (yes/no) scoring system, the assessment marks whether participants can produce an alternative explanation as an indicator of flexibility. Additionally, the EoE provides a conviction score (0-100%) that reflects the strength of the delusion and the inflexibility of such beliefs [26]. While the MADS and EoE have served as valuable tools in assessing inflexibility, their susceptibility to social desirability bias as self-report methods raised methodological concerns.

Recent research has advanced from assessing interpretation inflexibility using self-report measures and highlighted the importance of studying inflexibility in real-life, social situations. Specifically, cognitive tasks were developed to measure an individual’s bias against disconfirmatory evidence, a key mechanism underlying delusional beliefs in psychosis [37]. The Bias Against Disconfirmatory Evidence (BADE) task assessed how one forms neutral (i.e., non-emotional) interpretations about a statement. Later, the BADE assesses how one revises these interpretations, in light of disconfirming evidence [37]. An example of such statements is ‘‘Luke spends lots of time driving’’. The statement is presented along with four interpretations: (1) ‘‘Luke is a taxi driver’’ (lure); (2) ‘‘Luke is an ambulance driver’’ (lure); (3) ‘‘Luke is a fisherman’’ (absurd); and (4) ‘‘Luke delivers pizza’’ (true). With labels “Poor”, “Possible”, “Good”, and “Excellent” along a rating scale from 0 to 10, the participants then evaluate how plausible each of the four interpretations are for the given scenario [37]. Following this, a second statement is presented: ‘‘Luke is a young man’’. Finally, the statement ‘‘Luke’s car often smells like pizza’’ is presented, providing strong disconfirmatory evidence against both lure interpretations, and strong confirmatory evidence for the true interpretation. After the presentation of each new statement, participants are asked if they would like to revise their original ratings based on the new information. Inflexibility indices are calculated by extracting scores of “evidence integration” and “conservatism” from Principle Component Analysis [38]. Findings of the original BADE task indicated greater difficulties in individuals with schizophrenia (as compared to healthy controls) to revise their previously held interpretations of the scenario in light of disconfirmatory evidence [37]. Subsequent studies using the original BADE task have further confirmed that interpretation inflexibility is associated with the severity and persistence of delusions [25, 39].

Other tasks have measured inflexibility using various stimuli (e.g., reading verbal statements or viewing pictures). For example, picture completion tasks are designed to study how one responds to new visual information that disambiguates early beliefs about a picture [40]. Participants are presented with an incomplete picture (e.g., fishtail) and asked to choose if a description accurately depicts the picture (e.g., fish vs. mermaid). With additional information, participants are expected to revise their initial belief and draw a different, and more accurate, conclusion about the content of the picture (e.g., a mermaid) [40].

Utilizing these cognitive tasks, studies have started to explore the construct of inflexibility across different clinical populations. Research has linked inflexible interpretations of neutral (non-social; e.g., pictures of everyday objects) stimuli to the onset and maintenance of various psychiatric disorders, including depression, anxiety, PTSD, OCD, anorexia nervosa, psychosis and alcohol dependence [41–43]. However, emerging research has pointed to a more consistent association between psychopathology and inflexibility in *social* contexts, whereas findings are mixed in non-social situations. For example, people with social anxiety are more likely to demonstrate inflexibility in processing social, instead of non-social information (i.e., integrating negative information about others more readily, while resisting new positive information [44]. Another study showed persistent negative bias in anxious individuals despite obtaining positive social feedback from the experimenters in social situations [45]. Notably, the same study found no such inflexibility in the context of non-social information (shapes, colors), highlighting the specificity of inflexibility in social settings [45]. Indeed, as compared to the ability to revise interpretations of neutral stimuli (e.g., an everyday object), one’s ability to revise interpretations of social information (e.g., another person’s action) may be more closely associated with psychopathology. In the same vein, interpersonal difficulties and impaired social functioning are highly important in predicting psychopathology [46]. Exploring inflexibility in processing social information would further our understanding of social deficits implicated in psychopathology. Given that social interactions are essential to everyday life and to survival and that inflexibility significantly interferes with the processing of ambiguous social cues and information, it is vital to thoroughly investigate interpretation inflexibility within the social context [47].

While early tasks have provided a reliable measure of inflexibility during evidence integration, the stimuli used in these tasks are mainly pictures or statements that are neutral, non-self-referential, and do not involve much social context. These tasks may be helpful in exploring the basic cognitive mechanisms involved in evidence integration and belief revisioning (e.g., the visual processing of information, the ability to engage in evaluative reasoning). However, it remains unclear whether the flexibility measured by these tasks transfers to more complex contexts, such as social situations where individuals are expected to revise their interpretations of social stimuli.

## Assessing inflexibility in social information processing

To examine the effects of inflexibility in social situations, cognitive tasks are needed to recreate the social contexts in which interpretation ambiguity and subsequent needs for interpretation revisioning occur. In addition to utilizing cognitive tasks that provide social contexts to examine inflexibility, it is important to recognize that belief revisioning in social situations mostly take place in active affective states (i.e., when the individual who is performing the revisioning task is experiencing or processing emotions). Examining how an individual revises their existing interpretations when experiencing emotion (especially negative affect, as associated with persistent negative interpretation bias) might extend the ecological validity of flexibility research.

### The emotional BADE task

The emotional BADE task, developed based on the original (non-social) BADE task [37] is among the few measures that assesses both the initial bias and subsequent inflexibility involved in interpreting ambiguous social situations [15]. The emotional BADE task presents social scenarios depicting common interpersonal situations in gradual steps, prompting participants to revise their initial interpretations based on novel disconfirmatory information. Each scenario consists of three sentences, each revealing more information about the scenario. After each sentence, participants are asked to rate the plausibility of four interpretations on a scale from 1 (“Poor”) to 7 (“Excellent): one Absurd (remains implausible throughout the scenario), two Lures (initially most plausible but become less plausible on the second sentence of the scenario), and one True interpretation (initially less plausible than the Lures but becomes the most plausible by the end of the scenario). Two types of scenarios are presented in randomized order to examine whether interpretation inflexibility differs according to the valence of initial interpretations relative to that of the corresponding disconfirmatory evidence. The first type, disconfirming-the-negative scenarios, are designed to encourage initial negative interpretations resolved eventually by a positive ending. For example, one scenario reads as follows: “The company you are working for needs to lay off many employees. You are called in to see your boss” (sentence 1), “Your boss looks unhappy when you enter his office” (sentence 2), “Your boss shares how upset he is about having to lay off his employees, and states that he wants you to stay because of your collegiality and achievements” (sentence 3). In these scenarios, the two Lure interpretations are negative in valence (e.g., “Your boss wants you to leave the company because you’re not as good as the other employees.”, “The boss will have to let you go because you’re not a great fit with the team.”) and the True interpretation is positive in valence (e.g., “The boss wants to keep you in the company because you’re one of the better employees.”).

The second scenario type, disconfirming-the-positive scenarios, are initially positive but have a negative ending. For example: “Your best friends decide to go out for dinner on a Friday evening and ask you to join them” (part 1), “You arrive at the restaurant still wearing your casual work clothes” (part 2), “When you arrive, you friends say that you should not have come for dinner dressed like that” (part 3). In these scenarios, the two Lure interpretations have a positive valence (e.g., “Your best friends love it that you were able to join them for dinner”, “Your best friends think you look glamorous. ”) and the True interpretation has a negative valence (e.g., “Your best friends think you are not dressed properly”). The two scenario types are mixed and presented in randomized order across participants.

Emotional BADE task metrics are calculated based on the scoring method extracted from the confirmatory factor analyses. Negative interpretation inflexibility is calculated by taking the sum of plausibility ratings for the absurd statement at all three stages and those for the two lure statements at the third stage, minus the plausibility rating for the true statement at the third stage. Positive interpretation inflexibility is calculated by the same formula, plus the plausibility ratings for the first lure statement at the second stage and the true statement at the first stage. Positive interpretation bias is calculated by taking the sum of plausibility ratings for the true statement at all three stages in disconfirming-the-negative scenarios and the sum of plausibility ratings for the two lure statements at the first and second stages in disconfirming-the-positive scenarios. Negative interpretation bias is calculated by taking the sum of plausibility ratings for the true statement at all three stages in disconfirming-the-positive scenarios and the sum of plausibility ratings for the two lure statements at the first and second stages in disconfirming-the-negative scenarios. While the emotional BADE task measures interpretation inflexibility in processing social information [15, 16], the use of verbal statements may render the task taxing for participants with varying reading speed and comprehension ability. Subsequent research has developed a similar task using picture stimuli to capture interpretation inflexibility.

### The Interpretation inflexibility task

**Fig. 1:**
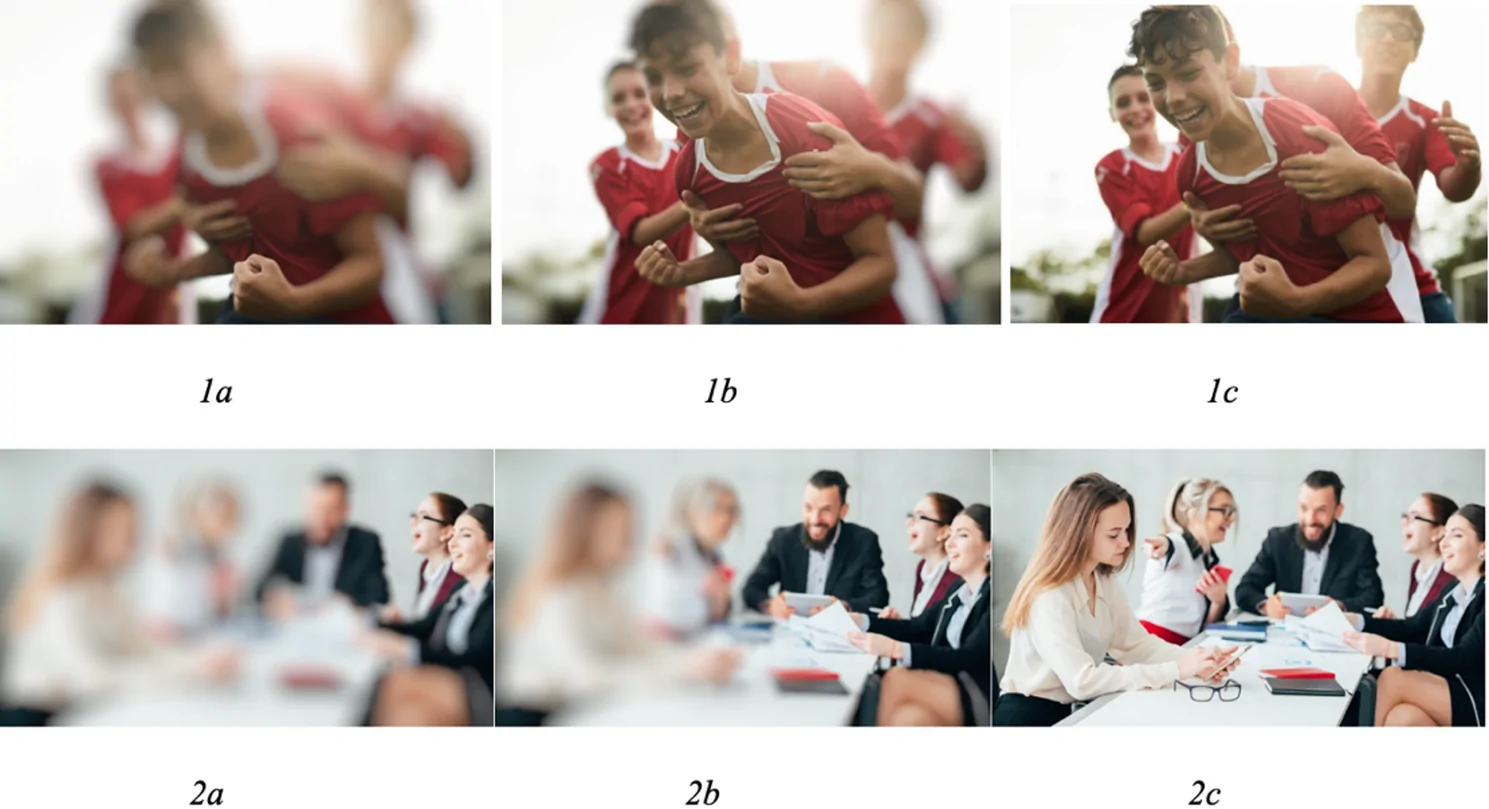
Examples of the IIT Scenarios

The Interpretation Inflexibility Task (IIT) is a similar paradigm that reliably captures inflexible bias in emotional social contexts. The IIT is a validated, picture-based task assessing individuals’ belief inflexibility and interpretation bias in social and emotional scenarios [9]. The IIT consists of 24 scenarios depicting interpersonal situations, each presented to the respondents in three stages of blurring. By gradually reducing the percentage of the photo that is blurred, the respondent receives more information that may help to resolve the initially ambiguous situation. Two types of scenarios are included in the IIT to examine whether interpretation inflexibility differs according to the valence of the new evidence in relation to the initial interpretation that was most strongly suggested by ambiguous stimuli. An example positive outcome scenario is shown in Fig. 1 (top), with each of the three pictures that make up the scenario (panels 1a-1c) revealing more information about the scenario, culminating in panel 1c to encourage a positive interpretation. An example negative outcome scenario of the IIT is also shown in Fig. 1 (panels 2a-2c). Each of the three pictures (panels 2a-2c) reveals more information about the scenario, culminating in panel 2c, which prompts a negative interpretation.

While both tasks enable the study of interpretation inflexibility within emotionally salient social contexts, they differ along key conceptual and methodological dimensions. The emotional BADE task primarily captures inflexibility in evidence integration, requiring individuals to revise initial interpretations in light of disconfirmatory information. In contrast, the IIT assesses inflexibility in ambiguity resolution, indexing the ability to converge on an accurate interpretation as stimuli become progressively clearer—a process of disambiguation rather than the revision of previously held beliefs [9].

These tasks also diverge in stimulus modality. The emotional BADE task relies on verbal scenarios, whereas the IIT employs visual stimuli in the form of gradually unblurring social scenes. This distinction carries important methodological implications. The verbal nature of the BADE task introduces potential variability related to reading and language comprehension, which may confound findings—particularly in cross-cultural contexts. By contrast, the IIT minimizes linguistic demands and may offer greater ecological validity, as ambiguous social information in everyday life is often encountered visually. Its reduced reliance on language and use of standardized affective imagery make it particularly well-suited for cross-cultural and community-based research.

Additionally, although the IIT still requires comprehension of response options, its pictorial presentation reduces confounds related to reading speed and verbal processing load. This feature may enhance its utility in neuroimaging contexts (e.g., EEG or fMRI), where minimizing extraneous cognitive demands is critical for isolating biobehavioral markers of interpretation bias and inflexibility [9].

That said, the emotional BADE task remains methodologically advantageous when the research question centers specifically on belief revision in response to disconfirmatory evidence. Because the IIT does not strongly instantiate an initially endorsed interpretation that is later violated, it may be less sensitive to this facet of inflexibility.

Taken together, both paradigms leverage socially relevant stimuli to probe flexibility in meaning-making. Given the centrality of social functioning to many forms of psychopathology, such tasks represent an important methodological advance in the study of interpretation processes.

### Psychopathology and inflexibility in social contexts

The aforementioned social interpretation tasks allow researchers to examine interpretation inflexibility as a separate construct in addition to interpretation bias, allowing for clarification of their unique associations with psychopathology.

## Associating inflexibility in social contexts with depression

Utilizing the emotional BADE task, researchers found that individuals exhibiting symptoms associated with depression are less likely to update negative interpretations even when presented with discrediting positive evidence. Advancing beyond the link between bias and depression, interpretation inflexibility in social situations is associated with cognitive patterns underlying depression, such as negative thinking, rumination, and feelings of sadness and hopelessness [15], hindering one’s ability to effectively adapt to changing situational demands [9]. It is also argued that interpretation inflexibility interferes with the implementation of emotion regulation strategies in depression, which might further prolong the experience of intense negative emotions [16].

Indeed, given the profound disruptions in social relationships among many with depression, social interpretation tasks are central and relevant in examining inflexibility as related to depressive symptoms. Studies using the emotional BADE task have found that, as social situations evolve with new information, inflexibility is manifested as people with depression continue to endorse interpretations that fit into their negative beliefs about the world and the self, while rejecting disconfirming evidence that carries a positive emotional valence [15]. Recent work has demonstrated that interpretation inflexibility in depression is specific to valence and displayed as a difficulty disengaging from negative interpretations, not the positive ones [15]. This finding adds to the existing evidence that in depression, persistent biases to negative affective stimuli make it significantly difficult to shift thinking from sad to happy content, thereby inhibiting interpretation flexibility across different social contexts. Feeding into the existing negative beliefs of the world, such inflexible negative interpretations of social situations may discourage individuals from future social interactions, extending low moods and social withdrawals in depression (Kube, 2023). Further, interpretation inflexibility of social stimuli is linked with depression even after accounting for the effect of negative bias on depression [15], highlighting the importance of examining inflexibility as a separate construct underlying depression, above and beyond the effect of negative bias.

Overall, recent research using social interpretation tasks highlights that inflexibility in depression is highly specific to valence, manifested as difficulties in revising negative beliefs and endorsing positive outcomes.

## Associating inflexibility in social context with psychosis

Social interpretation tasks provide a crucial extension from the previous research linking psychosis with inflexibility in interpreting neutral, non-social stimuli. Prior work has posited that belief inflexibility exists all along the severity continuum of psychosis [11]. People with psychotic-like experiences have demonstrated aberrant belief inflexibility in interpreting neutral, non-social stimuli [48]. Individuals who are at high risk for psychosis have reported similar interpretation bias and belief inflexibility in non-social situations [48]. On the more severe end of the spectrum, people with active schizophrenia are characterized by belief inflexibility or failures to revise initial neutral interpretations similar to that seen in subclinical individuals [49]. However, determining whether such inflexibility extends to social and emotional situations requires tasks such as the Emotional Bade and IIT. Given that psychotic symptoms and social functioning impairments are often co-occurring, pointing to poorer prognostic outcomes [33, 50], it is crucial to evaluate psychosis-related inflexibility in social situations, so as to provide a basis for improving early intervention and prevention efforts.

Different from the inflexibility driven by negative attentional bias and mood-congruent memory in depression, the inflexibility manifest among those suffering from psychosis may be a result of underlying cognitive deficits, including difficulties in the domains of attention and working memory. Numerous studies have highlighted such cognitive impairments as hallmark symptoms of psychosis. Research has shown that people with psychosis experience difficulty focusing their attention and avoiding distraction. In addition, jumping to conclusions, a cognitive pattern often observed in psychosis, is thought to be a manifestation or consequence of general cognitive deficits in individuals with psychotic symptoms [51].

The existing literature has established a connection between cognitive deficits and inflexibility in psychosis [49]. The theory of Dual Stream Modulation Failure suggests that, between the two streams of decision-making (i.e., automatic vs. deliberate), delusion-prone individuals are less likely to engage in analytic thinking, instead relying more on pre-existing beliefs to guide their information interpretation and belief formation [52, 53]. Difficulty engaging in analytic thinking and related bias in decision-making signal underlying cognitive deficits that drive the interpretation inflexibility in psychosis. Further evidence for such impairments in psychosis comes from the contexts in which such inflexibility exists. Prior work has shown that interpretation inflexibility in people with psychosis exists in both emotional and neutral contexts, suggesting that inflexibility is of a different origin than that in depression [15]. In addition, prior work has shown that people suffering from psychosis experience difficulties integrating disconfirming evidence to revise existing beliefs exist both in situations related to one’s delusional beliefs and in those that are irrelevant to one’s delusions [25]. Taken together, these findings confirm a distinct and fundamental cognitive deficit associated with psychosis, originating from the impaired ability to integrate evidence which drives interpretation inflexibility in psychosis, one that is independent of the emotional context.

While affect may seem less directly relevant in characterizing inflexibility in psychosis (than that in depression), stress reactivity and threat anticipation play a crucial role in the emergence and maintenance of psychosis [54], highlighting the importance of examining social contexts (where one’s stress responses and threat anticipation are often heightened). Prior work has shown that maladaptive appraisal of threat and difficulties regulating stress response may lead to selective attention to threat and interpretations of neutral stimuli as threatening [55]. Persistent hostile attributional bias, which refers to the rigidity in interpreting ambiguous social information as hostile, also underlies psychosis [56]. Neuroimaging research has further revealed a decreased activation in the ventral anterior cingulate cortex in response to anticipatory stress (i.e., stress during the anticipation of possible threat) [57]. The aberrant stress responding mechanism creates difficulties disengaging from an elevated state of anxiety, maintaining a continued anticipation of threat. Under such stress of anticipated threat, individuals are more likely to interpret ambiguous social stimuli as threatening, contributing to the maintenance of psychosis (e.g., persecutory beliefs, paranoia) and other stress-related symptoms [57].

## Discussion

This article reviewed research on interpretation updating in emotional, social contexts. Given that interpretation inflexibility research is closely connected with the negative bias literature in depression, whereas the inflexibility task development was informed by measures first developed in psychosis research, this review highlighted the role of interpretation inflexibility in depressive and psychotic symptoms. In particular, the paper underlined the clinical relevance of examining inflexibility in social information processing, noting important methodological advancement in cognitive task design that involves social situations. It is important to note that inflexibility in interpreting social situations may be a transdiagnostic mechanism underlying multiple clinical disorders, including but not exclusive to depression and psychosis mentioned in this review [43, 58]. A transdiagnostic perspective on inflexibility research can not only reveal the affective and cognitive mechanisms shared among different clinical experiences, but also uncover the specific factors underlying each unique symptom dimension. For instance, the interpretation inflexibility in depression is consistently found in social situations that require individuals to modify negative initial beliefs and endorse positive outcomes, whereas the association between difficulty endorsing negative outcomes and depressive symptoms is much less robust across studies [15, 16]. The specific inflexibility in disengaging from negative interpretations aligns with the negative attributional bias in depression – a cognitive tendency to attribute intrinsic, enduring, and global causes to negative experiences and events [59]. This suggests that cognitive aberrations underlying affective symptoms (e.g., depressive mood), such as negative attributional bias and rumination, may be context-dependent and sensitive to the emotional valence of social situations. Different from negative bias in depressive symptoms, paranoia is associated with a general inflexibility to disconfirm existing beliefs across positive, negative, and neutral social situations [60]. Such interpretation inflexibility exists above and beyond the impacts of initial interpretation bias, characterizing a sample with psychosis-proneness that requires interventions to improve executive functioning. To conclude, affective states and the social situations an individual is in provide important contextual information for understanding inflexibility in both depression and psychosis. Inflexibility in processing social stimuli may relate to the disrupted interpersonal processes (such as misattributing others’ intent as hostile or negative) in both psychosis and depression, relating to threat anticipation in psychosis and perpetuating social withdrawal in depression. Inflexibility is valence-specific in depression, as individuals experience greater difficulties disengaging from negative beliefs and/or endorsing positive information [15]. While inflexibility in psychosis exists regardless of the valence of the novel beliefs, affective contexts remain essential in understanding the aberrant stress responses and threat anticipation in psychosis. Taken together, it is integral to investigate not only the presence of interpretation bias, but also the persistence and inflexibility of such bias. Interpretation inflexibility as a standalone construct carries distinctive predictive values in explaining transdiagnostic symptoms.

It is worth noting that inflexibility exists across various stages of information integration, underlining the importance to be specific in future flexibility research (e.g., from selecting cognitive tasks to capture specific types of flexibility, designing the study to interpreting findings). This article focuses on inflexibility in social contexts. Specifically, inflexibility as discussed in this article refers to the rigidity and difficulty during belief revisioning, a process that involves revising initial interpretation bias, incorporating disambiguating information, and reaching a balanced understanding of social situations [16]. While other types of inflexibility are not reviewed in this article, it is important to recognize them as distinctive processes. For example, perseveration, as measured by the ability to switch from one task to another, (i.e., task-switching), represents a stereotyped response style during cognitive tasks [61]. Perseveration implicates executive functioning deficits that may or may not be related to the social settings. Psychological flexibility, as another example, is used to describe the resilience in adapting to new demands and coping with stressful situations [10]. While these forms of flexibility are central to psychological wellbeing and overall functioning, this article mainly focuses on evaluating the type of inflexibility arises from disambiguating social situations and revising prior interpretations. Given that inflexibility may show up in various aspects (e.g., as a cognitive deficit, during social interpretations, or in coping with stress), it is important for future studies to use more precise definitions when conducting research on inflexibility. Future research on interpretation inflexibility is also needed to investigate the specific cognitive process underlying the fixation on negative interpretations and the dismissal of positive disconfirmatory information. For example, physiological measures such as eye-tracking can provide additional information on whether people are fixating on the previously exposed negative stimuli or attending to the newly introduced negative information (Suslow et al., 2020).

Indeed, a better understanding of inflexibility, not only as a purely cognitive mechanism, but also as arising from motivational issues associated with emotional, social situations, can introduce novel intervention targets and improve treatment design. Existing interventions that encourage flexibility in clinical populations have targeted inflexible thinking styles in general. For example, cognitive restructuring in cognitive-behavioral therapy aims to identify ruminative thoughts and challenge inflexible beliefs about the self, the others and the world [62]. While these therapeutic practices can modify cognitive inflexibility, recognizing the interaction between affective states and inflexible thoughts may provide novel intervention targets (e.g., emotion regulation strategies may reduce distress and encourage flexibility). In addition, findings from the social psychology research on the general population can provide crucial background information for clinical scientists. For example, given that research has highlighted impression formation as a process affected by inflexibility [63], cognitive restructuring in clinical settings may aim at modifying biased first impressions in relationships. Further, it can be integral to consider one’s identity, cultural background and social group membership in conceptualizing clinical cases that involve inflexible thinking about social interactions and rigid beliefs about the self. Individuals experiencing minority distress, for example, may be more likely to rely on existing beliefs and assuming negative interpretations of social environments as self-preservation. Such contextual information may allow a holistic understanding of one’s inflexible beliefs and improve clinical decision-making, rendering cognitive strategies more applicable to individual situations. Last, while it is important to study how inflexibility *interferes* with one’s functioning in clinical presentations, understanding how inflexibility has served adaptive functions in the general population (e.g., increasing a sense of belonging, protecting group identity and maintaining social stability) can also further our knowledge about the motivators maintaining inflexibility over time.

Future research on inflexibility can benefit from not only utilizing tasks with interpersonal stimuli, but also conducting studies that incorporate interpersonal contexts. For instance, while hosting online survey studies is highly effective in gaining enough power and examining the general distribution of inflexibility, in-person studies incorporating emotional situations, such as one that exposes participants to acute stress, may be more ecologically valid in accurately capturing emotional responses and examining how individuals exhibit inflexibility in real-life social situations. It is also important to recognize that social information processing is a dynamic process, and that individuals encounter and integrate new evidence at different stages of their relationships. More fine-grained work is needed to further tease apart the nuances and determine the specific interpersonal processes disrupted by inflexibility (e.g., if inflexible bias occurs during the initial impression formation, or in the later stages of impression updating).

Taken together, existing research has revealed the interconnected roles of inflexibility and the impacted social processes in underlying a range of symptoms. Future work is needed to better elucidate the pathways between belief inflexibility in emotion-laden, social processes and individual symptom dimensions, so as to shed light on how inflexibility contributes to the development and maintenance of clinical disorders.

## Data Availability

Not applicable.
